# Three‐Year Follow‐Up of Neoadjuvant Tislelizumab with Chemotherapy in Locally Advanced Gastric and Gastroesophageal Junction Adenocarcinoma: Revealing Cancer‐Associated Fibroblast Heterogeneity Corresponding to PD‐1 Blockade Efficacy

**DOI:** 10.1002/advs.202508433

**Published:** 2025-12-01

**Authors:** Yao Lin, Xiong Sun, Chengguo Li, Ming Yang, Ke Wu, Ke Liu, Anshu Li, Xiaoming Shuai, Kailin Cai, Zheng Wang, Guobin Wang, Peng Zhang, Jianfeng Shen, Kaixiong Tao, Yuping Yin

**Affiliations:** ^1^ Department of Gastrointestinal Surgery Union Hospital Tongji Medical College Huazhong University of Science and Technology Wuhan Hubei Province 430022 China; ^2^ Department of Pathology Union Hospital Tongji Medical College Huazhong University of Science and Technology Wuhan Hubei Province 430022 China; ^3^ Department of Ophthalmology Ninth People's Hospital Shanghai JiaoTong University School of Medicine Shanghai 200025 China; ^4^ Institute of Translational Medicine National Facility for Translational Medicine Shanghai Jiao Tong University Shanghai 200240 China

**Keywords:** cancer‐associated fibroblasts, chemotherapy, locally advanced gastric cancer, neoadjuvant tislelizumab, SOX

## Abstract

The potential for immunotherapy in patients with locally advanced gastric cancer (LAGC) is recently confirmed. To report the 3‐year follow‐up data are aimed from a novel clinical trial. This is a prospective single‐arm phase II trial. Patients with LAGC received neoadjuvant tislelizumab plus SOX before surgery. Biopsies are obtained before treatment, and tumor samples post‐treatment underwent single‐cell RNA sequencing (scRNA‐seq). Spatial transcriptomics is conducted for validation. Cox regression models and Kaplan–Meier analysis are applied. A total of 49 patients are enrolled, and the median progression‐free (PFS) and overall survival (OS) are not achieved. The 3‐year PFS and OS rates are 64.1% and 73.2%, respectively. scRNA‐seq of 22, 248 cells from tumors from seven patients with LAGC enabled annotation of three cancer‐associated fibroblast (CAF) phenotypes. Spatial transcriptomics of gastric cancer samples validate the classification, showing inflammatory CAFs (iCAFs) negatively correlated with immunotherapy efficacy. Patients with LAGC show a favorable prognosis after neoadjuvant tislelizumab plus SOX treatment. CAF heterogeneity is crucial for patients with gastric cancer undergoing immunotherapy, and iCAFs is associated with an immunosuppressive microenvironment during PD‐1 blockade with SOX therapy and validated by in vitro experiments. Patients with LAGC with higher iCAF scores are resistant to SOX PD‐1 blockade.

## Graphical abstract

Heterogeneous cancer‐associated fibroblasts orchestrate dual immune microenvironments in LAGC.

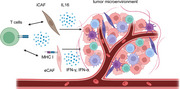

## 1 Introduction

The incidence of gastric cancer (GC) is rapidly increasing, and it is the third most common cause of cancer death globally.^[^ ^1^ ^]^ The prognosis of patients with locally advanced gastric cancer (LAGC) remains poor, with a 5‐year overall survival (OS) rate of only 38.7%.^[^ ^2^, ^3^ ^]^ Recent studies show immunotherapy, alone or when combined with chemotherapy, has promising antitumor activity and safety in patients with advanced GC.^[^ ^4^, ^5^ ^]^ Researchers are assessing the potential of immunotherapy in managing early‐stage GC during the perioperative period. Several Phase II clinical studies have revealed that neoadjuvant chemoimmunotherapy achieves considerable antitumor effectiveness in patients with resectable LAGC, with good rates of pathological complete response (pCR) and R0 resections.^[^ ^6^, ^7^, ^8^, ^9^, ^10^, ^11^, ^12^ ^]^ However, follow‐up data from the KEYNOTE‐585^[^ ^13^ ^]^ Phase III study show a 24‐month event‐free survival rate increase from 51% to 58% and OS increase from 69% to 72%, without significant differences. These results indicate the need for further research to improve immunotherapy accuracy and identify patients with LAGC who may benefit, with current research focusing on identifying predictive biomarkers to optimize immune checkpoint inhibitor effectiveness.^[^ ^14^, ^15^, ^16^ ^]^ Several biomarkers, such as PD‐L1 expression, tumor‐infiltrating lymphocytes, tumor mutational burden, and plasma‐based markers (cytokines and metabolites), have been investigated for their potential to enhance therapeutic responses.^[^ ^17^, ^18^, ^19^ ^]^ The complex characteristics of the tumor microenvironment (TME) and the intrinsic diversity of tumors remain poorly understood. Exploring the role of tumor‐infiltrating cells within the LAGC TME is vital for enhancing treatment.

Research^[^ ^20^, ^21^ ^]^ suggests that cancer‐associated fibroblasts (CAFs) constitute a heterogeneous and dynamic population involved in extracellular matrix (ECM) remodeling, modifying metabolic pathways, immune cell exclusion, and drug resistance.^[^ ^22^, ^23^ ^]^ CAFs can either facilitate or inhibit tumor growth, making them vital targets for cancer therapy. Generally, CAFs are categorized into three subtypes based on their functional roles: myofibroblastic CAFs (myCAFs), inflammatory CAFs (iCAFs), and extracellular matrix‐associated CAFs (eCAFs).^[^ ^24^, ^25^ ^]^ The functions of CAF immunotherapy remain complex and not fully elucidated. Ma et al.^[^ ^26^ ^]^ reported that iCAFs contribute to establishing an immunosuppressive microenvironment in patients with breast cancer (BRCA) undergoing anti‐PD‐1 immunotherapy. Another study focusing on hepatocellular carcinoma with immunotherapy found that CAFs interact to promote a tumor immune barrier (TIB) structure formation and limit tumor immune infiltration.^[^ ^27^ ^]^ Distinct CAF subsets play functional roles in modulating the immunoregulatory milieu in human head and neck cancer.^[^ ^28^ ^]^ However, the specific functions of CAF subsets in LAGC, their distinct contributions to the TME, and their responses to neoadjuvant therapy remain insufficiently explored.

Here, a novel clinical trial was conducted using neoadjuvant tislelizumab with SOX chemotherapy in LAGC, and the TME landscape was examined using scRNA‐seq of samples involved in the WuhanUHGI001 trial and the GEO dataset (GSE268238).

## 2 Results

### 2.1 Patient Characteristics

Forty‐nine patients were enrolled. Patient baseline characteristics are summarized in **Table** **1**. The median age was 58.5 (range: 34–74); 39 (79.6%) were male, and 12 (24.5%) had primary tumors located at the gastroesophageal junction. Baseline clinical tumor stage cT3 and clinical node stage N+ were observed in 27 (55.1%) and 49 (100.0%) patients, respectively. Among the 46 (93.9%) patients assessed for PD‐L1 expression, 29 (59.2%) had a combined positive score ≥1. Most tumors (47/49, 95.9%) were mismatch repair‐proficient (pMMR). All patients completed neoadjuvant therapy; two completed two cycles of neoadjuvant therapy. One patient experienced disease progression after two cycles, and one initially showed suspected disease progression upon imaging assessment after two cycles; however, the final diagnosis via surgical evaluation was pseudoprogression. Two patients (2/49) had unresectable lesions. Forty‐seven patients underwent gastrectomy with D2 lymphadenectomy, 46 received adjuvant therapy, and one (1/47) did not receive adjuvant therapy because of postoperative death.

**Table 1:** Patient characteristics.

Characteristics	Total [n = 49], n [%]
Age, years, median (range)	58.5 (34–74)
Sex, n (%)	
Male	39 (79.6)
Female	10 (20.4)
ECOG PS, n (%)	
0	40 (81.6)
1	9 (18.4)
Clinical T stage, n (%)	
3	27 (55.2)
4	21 (43.8)
Clinical N stage, n (%)	
1	31 (63.3)
2	15 (30.6)
3	3 (6.1)
Tumor location	
Pylorus	5 (10.2)
Cardia	12 (24.5)
Fundus	13 (26.5)
Body	19 (38.8)
Bormann subtype, n (%)	
I	9 (18.4)
II	24 (49.0)
III	13 (26.5)
IV	3 (6.1)
Lauren's classification, n (%)	
Diffuse type	30 (61.2)
Intestinal type	16 (32.7)
Mixed type	3 (6.1)
Signet ring cell component, n (%)	
Presence	10 (20.4)
Absence	39 (79.6)
PD‐L1 CPS, n (%)	
< 1	17 (34.7%)
1–4	11 (22.4%)
5–10	9 (18.4%)
≥ 10	9 (18.4%)
Missing	3 (6.1%)
Her‐2 score, n (%)	
0	30 (61.2%)
1	12 (24.5%)
≥ 2	7 (14.3%)
MSI/MMR, n (%)	
MSI‐H/dMMR	2 (4.1%)
MSS/pMMR	47 (95.9%)
Neoadjuvant therapy cycle	
2	2 (4.1)
3	47 (95.9)
Histological grade	
Poorly differentiated	33 (67.3)
Moderately differentiated	16 (32.7)

Abbreviations: CPS, combined positive score; ECOG, Eastern Cooperative Oncology Group performance score.

### 2.2 Safety and Tolerability

TRAEs occurred in 32 (65.3%), 35 (74.5%), and 33 (71.7%) patients during the neoadjuvant, postoperative, and adjuvant periods, respectively. Grade 3–4 TRAEs were observed in six patients (12.2%) during the neoadjuvant period, eight patients (17.0%) during the postoperative period, and seven patients (15.2%) during the adjuvant period. The most frequent TRAEs during the neoadjuvant period included myelosuppression, white blood cell decreased, anemia, neutropenia, and vomiting, while myelosuppression, white blood cell count decreased, neutropenia, platelet count decreased, and vomiting were most common during the adjuvant period.

Overall, most TRAEs were Grade 1–2 and manageable with supportive care. Detailed data are presented in Table S1 (Supporting Information). The most frequent irAEs were rash, which was generally mild (Grade 1–2). However, one patient experienced fatal immune‐related toxicity, diagnosed as hemophagocytic lymphohistiocytosis (HLH) during the study period.

### 2.3 Pathological and Follow‐Up Results

**Figure 1:**
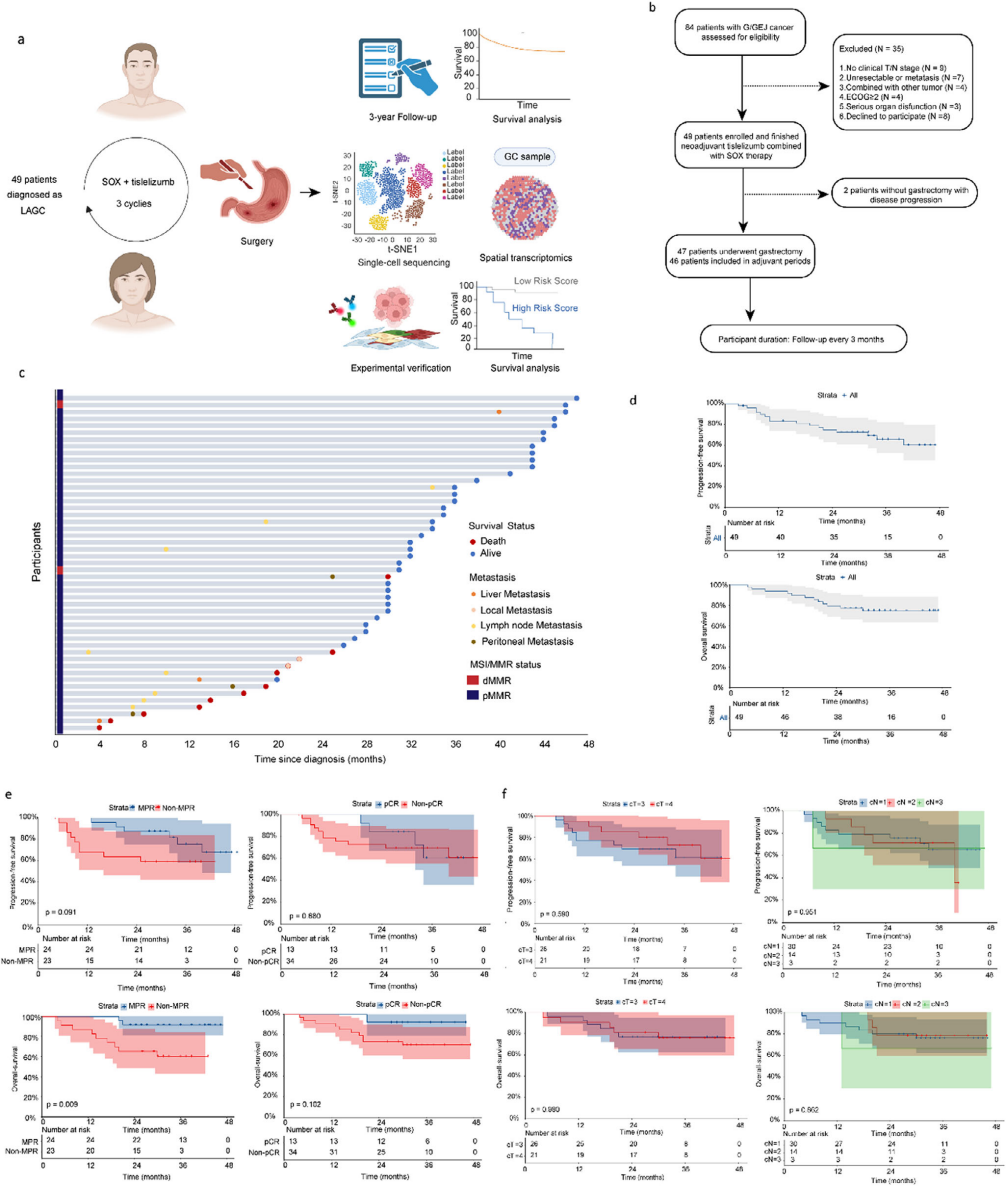
Research design and results of clinical trials. a) Study Design. b) Flow chart. c) Swimming plot showing events during treatment and follow‐up (n = 47) d) Kaplan‐Meier analyses of PFS and OS of all patients in this cohort; e) Kaplan‐Meier analyses of PFS and OS of patients with pCR and MPR; f) Kaplan‐Meier analyses of PFS and OS of patients stratified by different TN stages. PFS, progression‐free survival; OS, overall survival; pCR, pathological complete remission; MPR, major pathologic response.

Thirteen (26.5%) patients achieved pCR, 24 (49.0%) achieved MPR, and 47 (95.9%) underwent R0 resection. In the surgical population, the pCR, MPR, and R0 resection rates were 27.7% (13/47), 51.1% (24/47), and 100% (47/47), respectively. Two patients (2/47, 4.3%) who achieved a pCR were mismatch repair‐deficient (dMMR) (**Figure** **1**c). pCR and MPR rates remained not significantly different, while pCR rates of patients with dMMR were significantly different from those of pMMR patients (χ^2^ = 5.774, P = 0.016) (Table S2, Supporting Information). The median follow‐up period was 37 months (quartiles: 31–43 months). Median PFS and OS were not reached. The 3‐year PFS and OS rates were 64.1% and 73.2%, respectively (Figure 1d). PFS (P = 0.680) and OS (P = 0.102) were not significantly different between the pCR and non‐pCR groups. However, PFS (P = 0.091) and OS (P = 0.009) were significantly higher in the MPR group than those in the non‐MPR group (Figure 1e). PFS and OS were not significantly different between different TN stages (Figure 1f).

### 2.4 Identification of CAFs in LAGC under PD‐1 Blockade Treatment

**Figure 2:**
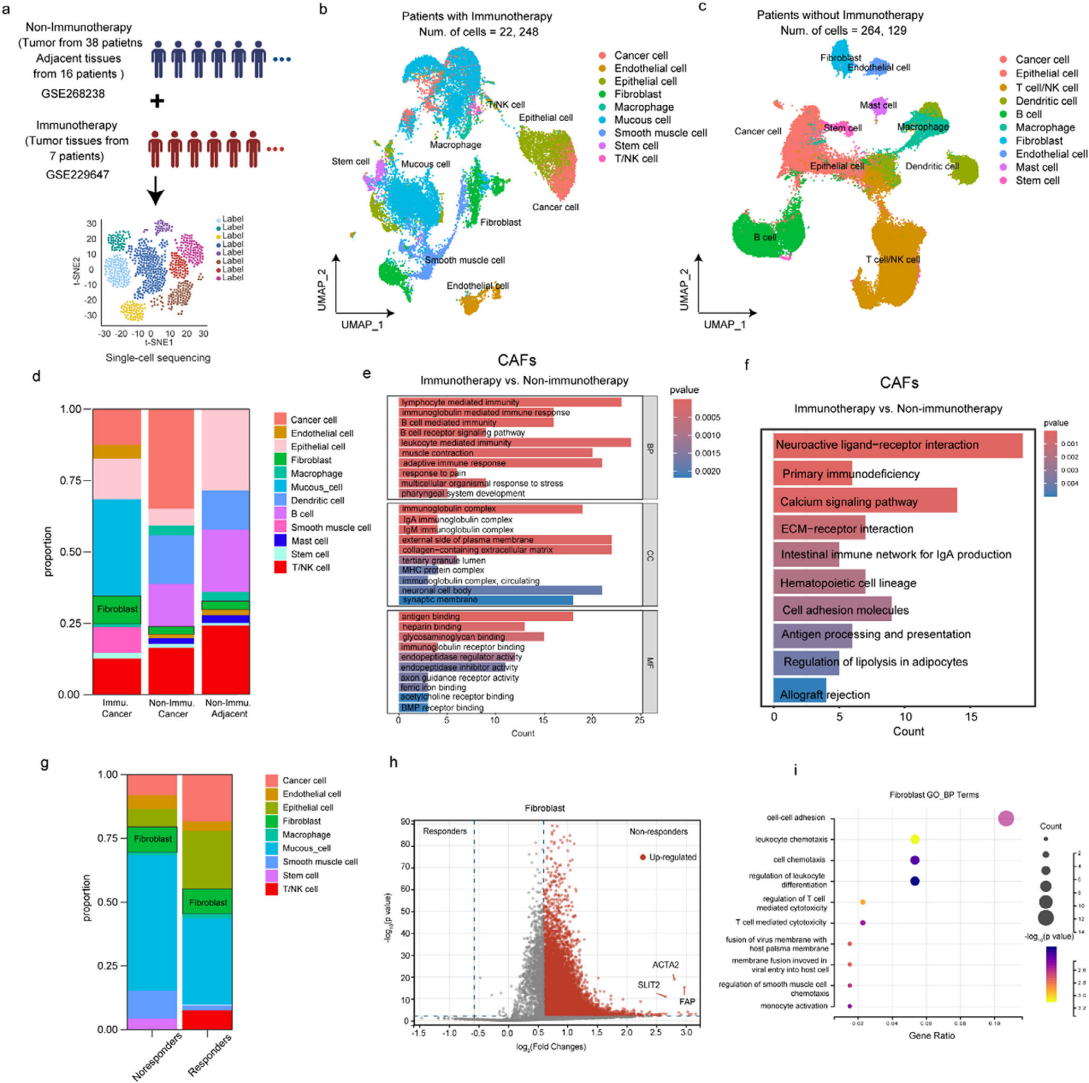
CAFs were responsive to immunotherapy in LAGCs. a) Schematic illustration of the study design and datasets used for single‐cell sequencing. b) UMAP visualization of cell clusters in patients who received immunotherapy. c) UMAP visualization of cell clusters in patients without immunotherapy. d) Proportions of different cell subtypes across immunotherapy, non‐immunotherapy, and adjacent tissues. e) GO and KEGG enrichment analyses of differentially expressed genes in CAFs between immunotherapy and non‐immunotherapy groups. f) Top enriched signaling pathways of CAFs comparing immunotherapy and non‐immunotherapy groups. g) Proportions of cell subtypes between responders and non‐responders. h) Volcano plot showing differentially expressed genes in fibroblasts between responders and non‐responders. i) GO‐BP enrichment of fibroblast‐related genes in non‐responders. GO‐BP, gene ontology–biological process; CAF, cancer‐associated fibroblast.

**Figure 3:**
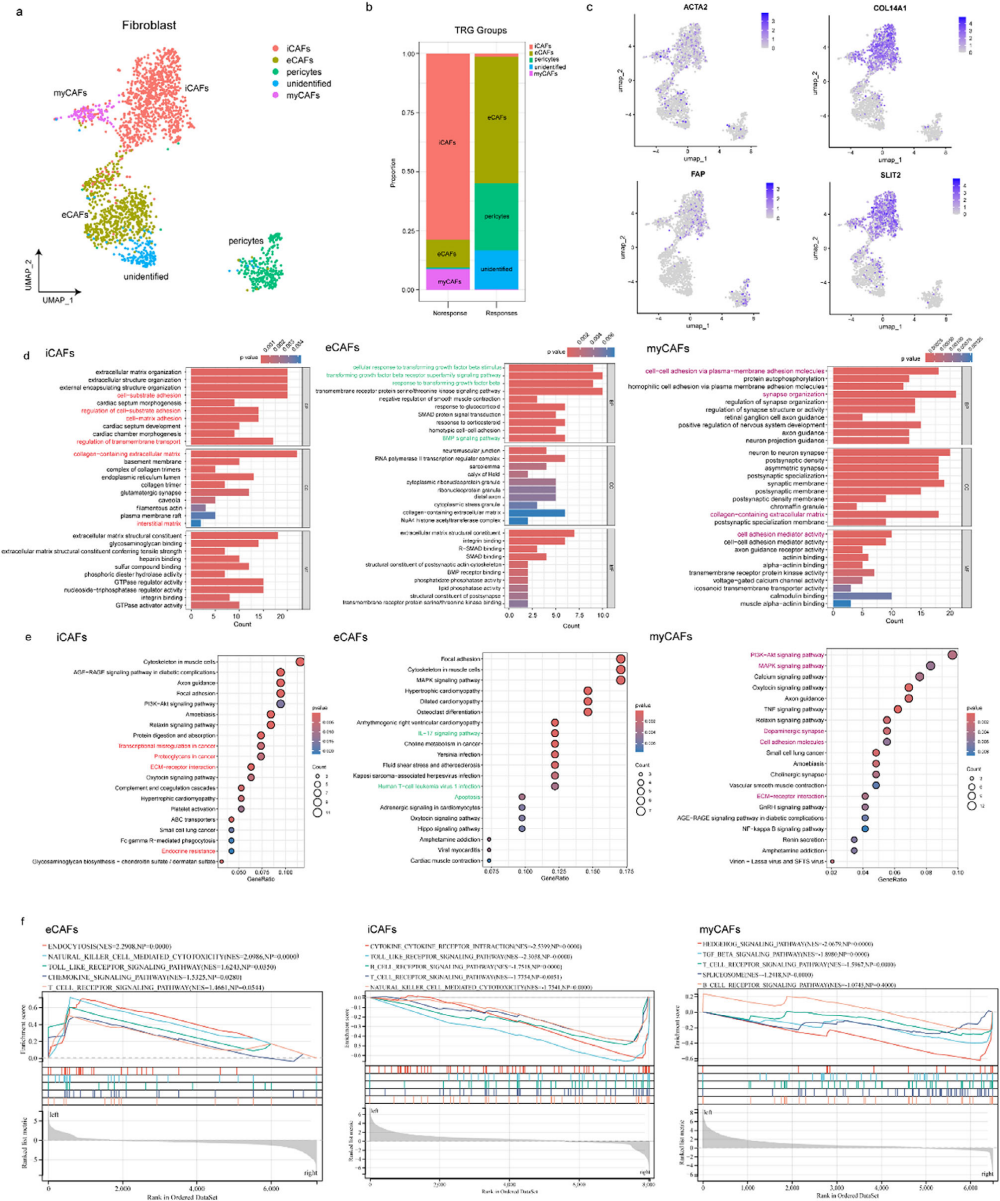
eCAFs and iCAFs showed different reactions to immunotherapy. a) UMAP visualization of CAF subtypes identified in LAGC tissues. b) Proportions of CAF subtypes in responders and non‐responders. c) UMAP feature plots showing the expression levels of representative marker genes (ACTA2, COL14A1, FAP, and SLIT2) in CAF subtypes. d) GO functional enrichment analysis of iCAFs, eCAFs, and myCAFs. e) KEGG pathway enrichment analyses of iCAFs, eCAFs, and myCAFs. f) GSEA of iCAFs, eCAFs, and myCAFs. GO, gene ontology; KEGG, Kyoto Encyclopedia of Genes and Genomes; GSEA, gene set enrichment analysis; CAF, cancer‐associated fibroblast; iCAF, inflammatory CAF; eCAF, extracellular matrix‐related CAF; myCAF, myofibroblastic CAF; LAGC, locally advanced gastric cancer.

To identify and classify CAF types, we analyzed an scRNA‐seq dataset from 7 human LAGC specimens in our cohort and 38 human GC specimens in GSE268238 (**Figure** **2**a); the clinicopathological baseline of our cohort is shown in Table S3 (Supporting Information). Cell clusters in scRNA‐seq of patients with immunotherapy and non‐immunotherapy were identified (Figure 2b,c; Figure S1a,b, Supporting Information). The proportion of CAFs in patients with immunotherapy was higher than in patients with non‐immunotherapy (Figure 2d; Figure S1c, Supporting Information). Further, cell clusters in responders and non‐responders were identified (Figure S1d,e, Supporting Information). We identified 11718 fibroblasts (2,187cells for patients with immunotherapy) based on unsupervised clustering (Table S4, Supporting Information). Meanwhile, immune‐related pathways were notably enriched in CAFs from immunotherapy‐treated gastric cancer patients (Figure 2e,f). Although the proportion of CAFs remained unchanged between responders and non‐responders (Figure 2g), gene expression was up‐regulated in CAF clusters in non‐responders (Figure 2h; Table S5, Supporting Information), primarily in cell‐cell adhesion and T‐cell regulation (Figure 2i **)**. Of these, we identified 277 cells as pericytes based on COL1A1 and MYH9 expression, resulting in 1750 CAFs (**Figure** **3**). This dataset was used to investigate fibroblast phenotypic heterogeneity in LAGC treated with immunochemotherapy.

After batch correction, we performed unsupervised hierarchical clustering of the single‐cell gene expression profiles of all stromal cells, identifying eight clusters (Figure S2a, Supporting Information) at a resolution of 0.4 (selected to balance overclustering and retain the ability to identify rare cell types). We examined the top differentially expressed genes for each cluster (Table S6, Supporting Information). In two cases, we manually merged clusters with highly overlapping functionally related differentially expressed genes (0, 2, and 5 as iCAF, and 1 and 4 as eCAF), resulting in five CAF cell types (Figure 3a). The proportion of iCAF in non‐responders was higher than that in responders (Figure 3b). Based on these analyses, we classified the clusters into five CAF types (iCAFs, eCAFs, myCAFs, pericytes, and unidentified subtypes), and focused on the three main CAF subtypes (iCAFs, eCAFs, and myCAFs).

iCAFs were the largest cluster (n = 989), characterized by the unique expression of a phospholipase encoded by SLIT2 and genes involved in the complement pathway (such as CFD and CD34) (Figure 2c,d). CD34, a marker for hematopoietic stem cells expressed by fibroblasts and linked to inflammation, was among the top differentially expressed genes in this cluster. iCAFs also showed high expression of complement protein, including C3 and C7 (Figure 3a). CFD, COL14A1, FAP, and SLIT2 expression was higher in iCAFs (Figure 3c,f; Figure S3b, Supporting Information). The ECM‐receptor interaction pathway, Fcγ R‐mediated phagocytosis, and endocrine resistance were also strongly enriched in iCAFs (Figure 3d,e).

Two clusters of eCAFs (clusters 4 and 1) were identified, characterized by high expression of genes encoding matrix proteins, particularly the noncollagenous matrix proteins POSTN and CXCL14 (Figure 2c,d), which were combined into a single cluster. The resulting CAFs (n = 652) expressed collagen‐encoding mRNAs (COL4A5 and COL6A2). mRNAs encoding noncollagenous matrix proteins (POSTN) and mucoproteins (MUC1 and MUC5AC) were among the top 20 differentially expressed genes in this group (Figure S3c, Supporting Information). eCAFs also expressed high levels of genes associated with migration (POSTN and CXCL14) (Figure S3d, Supporting Information). The top two differentially expressed genes in this cluster were CXCL14 and POSTN. Despite the strong overlap in their top differentially expressed genes, the two merged clusters (4 and 1) may still represent distinct subgroups of eCAFs. We performed GO/KEGG pathway enrichment analysis using hallmark pathways to validate our annotations in an orthogonal manner. For eCAFs, upregulated GO terms included transforming growth factor beta stimulus, and upregulated KEGG pathways included the IL‐17 signaling pathway, associated with activated myCAF development, human T‐cell leukemia virus 1 infection, and apoptosis (Figure 3d,e). These genes, associated with matrix remodeling and migration, further support our eCAF annotation.

For myCAFs, cluster 7 was characterized by markedly high expression of gene ACTA2 (α‐SMA) (Figures 2c and 3b). Additionally, we performed GO/KEGG pathway enrichment analysis, finding ECM−receptor interaction, PI3K−Akt signaling, and TNF signaling were strongly enriched in myCAFs. Cell adhesion activity and nervous system regulation were also enriched in myCAFs (Figure 3d,e).

We also conducted GSEA of iCAFs, eCAFs, and myCAFs. Immune pathways such as natural killer cell receptor (NES = 2.099, P<0.001) and T‐cell receptor (NES = 1467, P = 0.05) signaling were significantly upregulated in eCAFs, whereas T‐cell receptor (NES = −1.776, *p* = 0.005), B cell receptor (NES = −1.752, *p* < 0.001), and natural killer cell receptor signaling pathways (NES = −1.754, *p* < 0.001) were significantly downregulated in iCAFs; T‐cell receptor signaling (NES = −1.597, *p* < 0.001) was also significantly downregulated in myCAFs (Figure 3g,h; Figure S2e–g, Supporting Information). Based on these analyses, we hypothesized that eCAFs promoted immune activation while iCAFs and myCAFs promoted the formation of an immunosuppressive environment in LAGC.

### 2.5 Pseudotime Analysis and Interactions between CAFs with T‐cells

**Figure 4:**
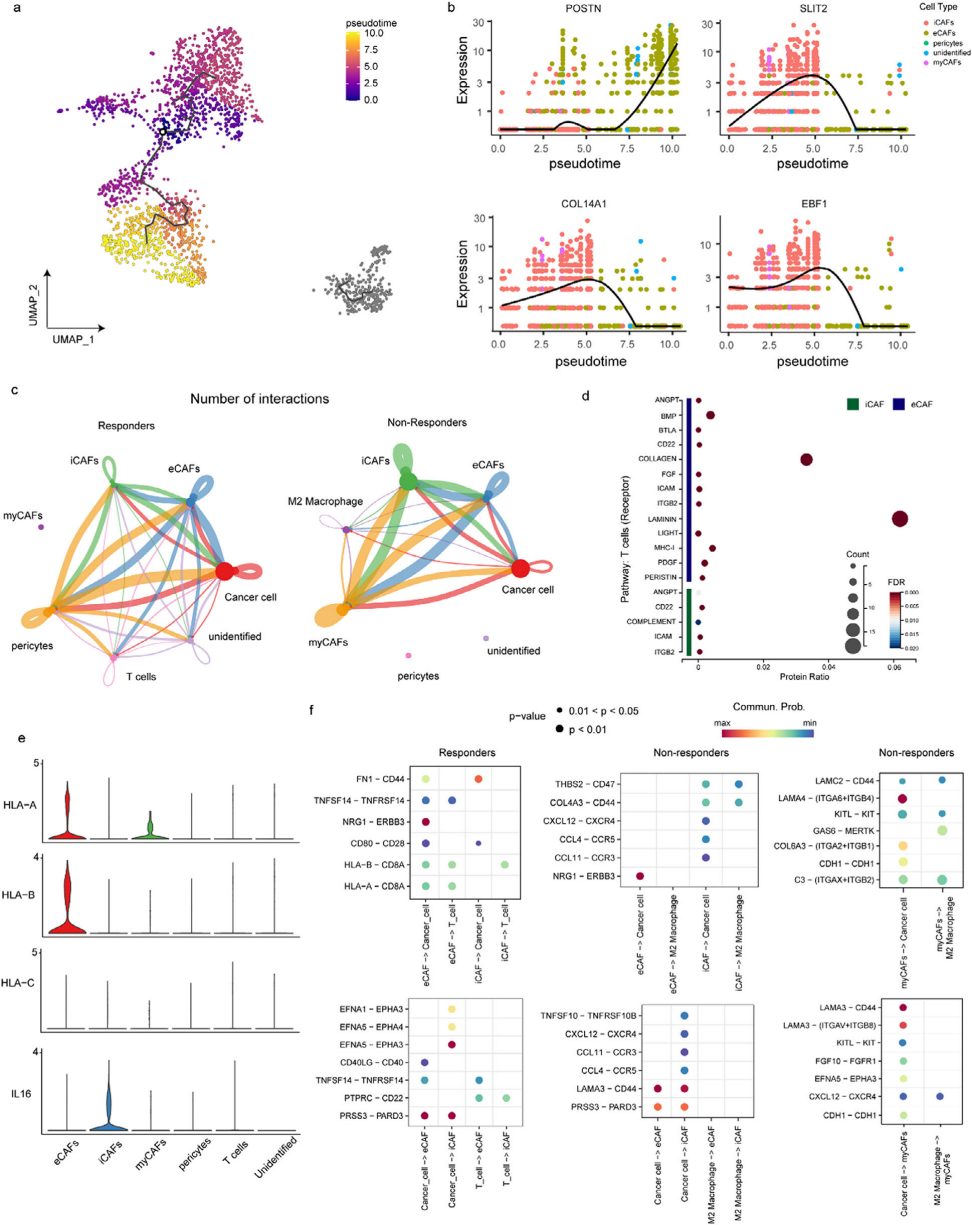
eCAFs and iCAFs interact with T cells in immunotherapy. a) Pseudotime trajectory analysis of CAFs illustrating the differentiation relationship between iCAFs and eCAFs. b) Expression dynamics of representative genes (POSTN, SLIT2, COL14A1, and EBF1) along pseudotime. c) Interactions between different CAF subtypes and other cell clusters in responders and non‐responders. d) Detailed interactions in different CAF subtypes and T‐cells. e) Expression of HLA‐A, HLA‐B, HLA‐C, and IL16 markers in CAF subtypes and T‐cells. f) Comprehensive ligand–receptor interaction network between different CAF subtypes and other immune or stromal cells. CAF, cancer‐associated fibroblast. iCAF, inflammatory CAF; eCAF, extracellular matrix‐related CAF.

Pseudotime analysis showed iCAFs could change into eCAFs (**Figure** **4**a) during PD‐1 blockade treatment, and that POSTN, SLIT2, COL14A1, and EBF1 could be important biomarkers (Figure 4b).

Immunosurveillance escape is a major hallmark of cancer; CAFs facilitate this by providing a physical barrier and impacting the immune TME. The interaction counts between eCAFs and other subclusters were higher than those between iCAFs and myCAFs in responders (Figure 4c), highlighting their potential immunoregulatory role with more interactions with T‐cells (Figure 4d). Shared and specific reciprocal communication was observed between different CAFs and cells. CD44, an important index for T‐cell proliferation and navigation in antitumor immunity, exclusively interacts with FN1, COL4A4, and COL4A3 in iCAFs among cancer cell subclusters. As another important index, the expression levels of major histocompatibility complex (MHC) class I molecules such as HLA‐A and HLA‐B were significantly higher in eCAFs, whereas MHC II molecules such as CD74, HLA‐DRB1, HLA‐DRA, HLA‐DQA1, and HLA‐DPA1 showed no significant difference (Figure 4a). IL16 was highly expressed in iCAFs, interacting with CD4^+^ T‐cells (Figure 4e). In addition, iCAFs could release more chemokines such as CCL4, CCL11, and CXCL12 in non‐responders. HLA‐A and HLA‐B in eCAFs interacted with CD8A in T‐cells, an important marker of cytotoxic T‐cells; LAMC, LAMA4, LAMA3, and CDH1 in myCAFs interacted with CD44, ITGA6, ITGB4, and CDH1 in cancer cells, which are important markers for tumor proliferation (Figure 4f). Detailed ligand‐receptor interactions are shown in Figure 4b,c.

### 2.6 eCAF and iCAF Involved in Different Immune Microenvironments

**Figure 5:**
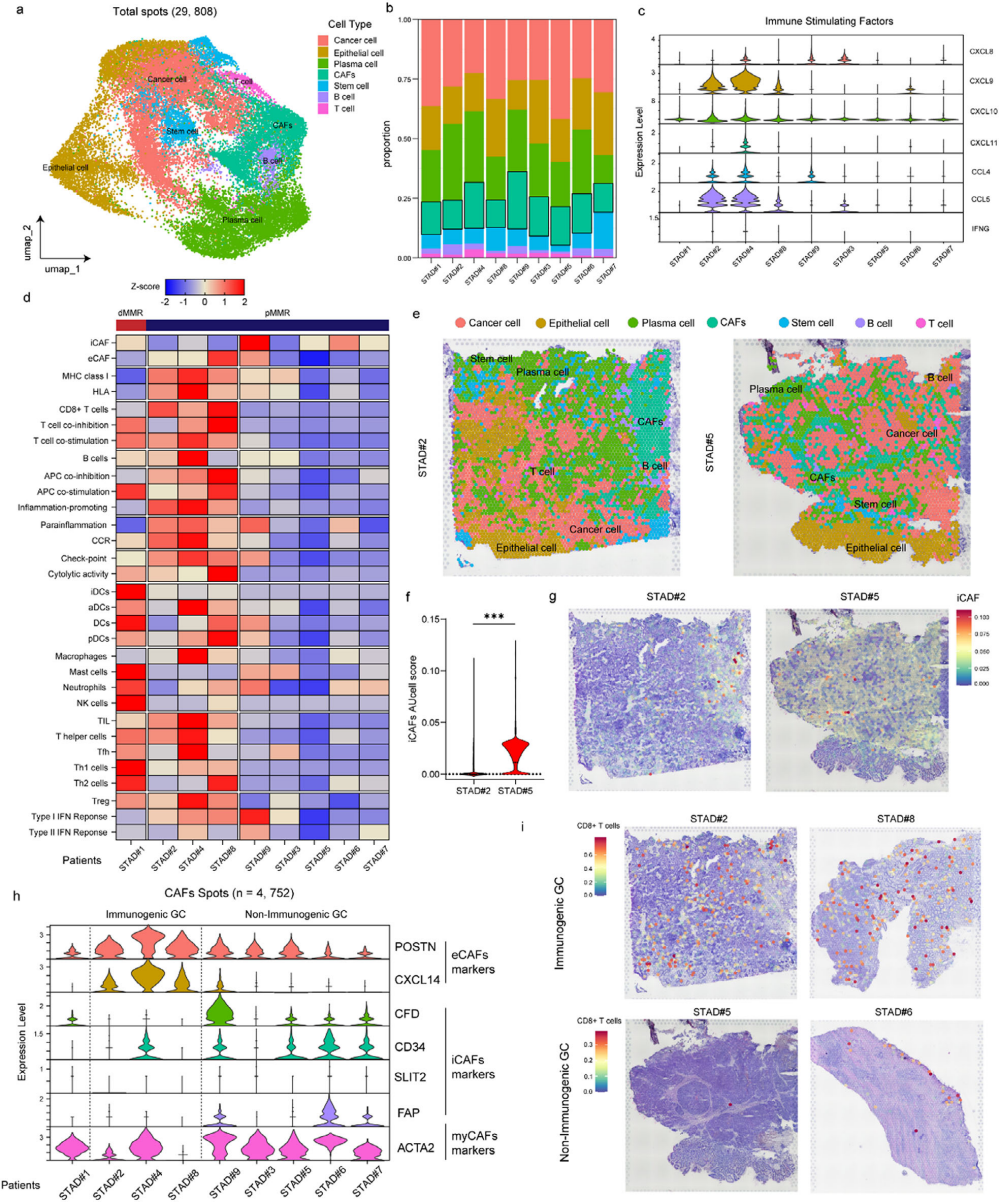
The different CAF subtypes involved the immune microenvironment. a) UMAP of all spots (n = 29808) from nine GC primary samples. b) The proportion of cell types in all samples. c) Violin plots showing the expression of immune‐stimulating factors CXCL8, CXCL9, CXCL10, CXCL11, CCL4, CCL5, and IFNG. d) Heatmap for AUcell scores of immune pathways and CAF subtypes (iCAFs and eCAFs) across GC samples. e) The spatial representation of cell type niches in STAD#2 (immunogenic GC) and STAD#5 (non‐immunogenic GC). f) The expression level of iCAFs in spots of STAD#2 and STAD#5. g) The spatial representation of iCAFs in spots of STAD#2 and STAD#5. h) Violin plots for the expression of POSTN, CXCL14, CFD, CD34, SLIT2, FAP, and ACTA2 in all GC samples. i) The spatial representation of CD8+ T cells in spots of STAD#2, STAD#8, (immunogenic GC) STAD#5, and STAD#6 (non‐immunogenic GC). ^*^ *p* < 0.05, ^**^ *p* < 0.01, ^***^ *p* < 0.001. CAF, cancer‐associated fibroblast; iCAF, inflammatory CAF; eCAF, extracellular matrix‐related CAF; GC, gastric cancer.

Spatial transcriptomics analyses of nine LAGC specimens yielded spatially resolved expression profiles, with a total of 29808 spots (median: 3491 spots) from GSE251950 (**Figure** **5**a; Figure S5a, Supporting Information). Baseline information is shown in Table S7 (Supporting Information). Using an scMC processing and a clustering pipeline, the spots were grouped into 15 clusters based on their gene expression profiles and were merged into seven cell types based on conventional marker genes (Figure S5b, Supporting Information). Cell‐type proportions for each spot were predicted using markers from the above cohort (Figure 5b). We checked the expression of ordinary immune factors such as CCL4, CCL5, CXCL8, CXCL9, and CXCL10, and found they were much higher in STAD#2, STAD#4, and STAD#8 (Figure 5c). We calculated the activation score (Figure 5f) of immune pathways, iCAF, and eCAF (gene lists shown in Table S8, Supporting Information), finding that the immune pathways were activated in STAD#2, STAD#4, and STAD#8 while they remained inactive in STAD#9, STAD#3, STAD#6, and STAD#7 (Figure 5d). We selected STAD#2, STAD#4, and STAD#8 as immunogenic GC and STAD#9, STAD#3, STAD#6, and STAD#7 as non‐immunogenic GC. In all nine samples, visualization of the spatial localization of the spots for each cluster was correlated to histologic features (Figure 5e; Figure 5c).

**Figure 6:**
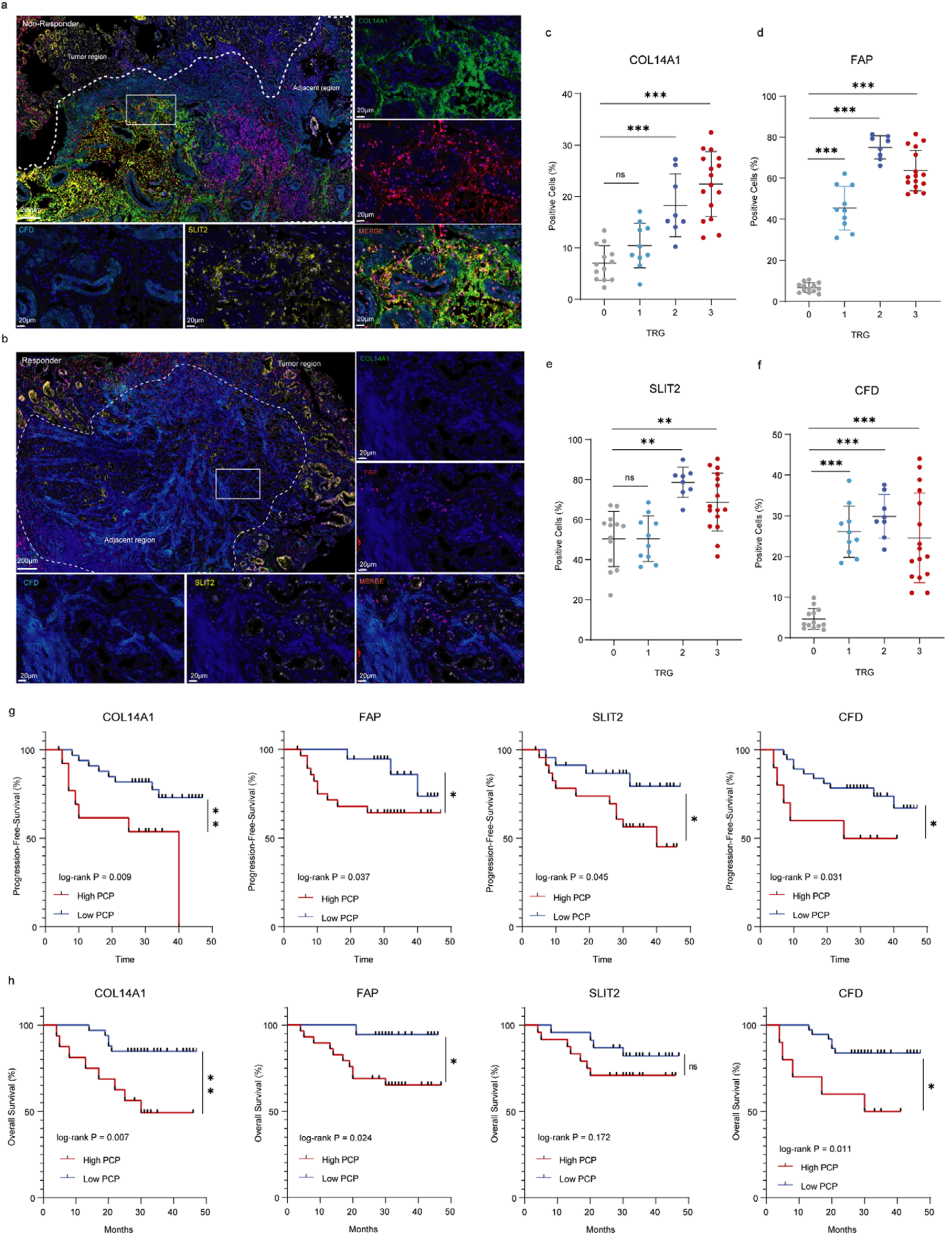
Multi‐IF confirmed the duality of CAFs in immunotherapy. a,b) Multiplexed immunofluorescence staining of FAP, SLIT2, CFD, and COL14A1 in LAGC tissues of responders and non‐responders treated with immunotherapy. Multiplexed immunofluorescence assays were performed twice on tumor samples following assay optimization. c–f) Quantification of PCP for COL14A1, FAP, SLIT2, and CFD in patients with LAGC stratified by TRGs. g) Kaplan‐Meier analysis of PFS based on different expression levels of COL14A1, FAP, SLIT2, and CFD in patients receiving immunotherapy. h) Kaplan‐Meier analysis of OS according to the expression levels of COL14A1, FAP, SLIT2, and CFD in patients receiving immunotherapy. PCP, percentage of positive cells; TRG 0, (complete pathological response in the primary tumor and lymph nodes, no residual tumor cells), TRG 1, (near‐complete response, ≤10% residual tumor cells), TRG 2, (partial response, 10–50% residual tumor cells), and TRG 3, (poor or no response, ≥50% residual tumor cells). PFS, prognosis‐free survival; OS, overall survival; ^*^ *p* < 0.05, ^**^ *p* < 0.01, ^***^ *p* < 0.001.

Based on the expression of the top 30 marker genes of iCAFs and eCAFs in scRNA‐seq results (**Figure** **6**a), we calculated the Z‐score of iCAFs and eCAFs. iCAFs were highly expressed in non‐immunogenic GC, while eCAFs were highly expressed in immunogenic GC (Figure 5d; Figure S6b, Supporting Information). We selected STAD#2 and STAD#5 as examples for visualization of the spatial localization of the iCAF score, and enrichment was obvious in STAD#5 compared with STAD#2 (Figure 5f,g). Corresponding to stromal tissue areas with high collagen gene expression and markers found in scRNA‐seq (POSTN, CXCL14, CFD, CD34, FAP, SLIT2, and ACTA2), eCAF markers were highly expressed in immunogenic GC, while iCAF and myCAF markers were highly expressed in non‐immunogenic GC (Figure 5h). The CD8+ T‐cell score (Figure 5i) of spots in immunogenic GC (such as STAD#2 and STAD#8) was much more enriched than that of non‐immunogenic GC (such as STAD#5 and STAD#6). The MHC class I pathway (Figure S6c,d, Supporting Information) was highly activated in STAD#2 compared with STAD#5, and eCAFs had a similar distribution to MHC class I (Figure S6e,f, Supporting Information). These results confirmed that iCAFs could inhibit immune cell infiltration while eCAFs promoted it through the MHC class I pathway in GC.

### 2.7 iCAF and eCAF Infiltration Score Predicted PD‐1 Blockade Treatment Efficacy

We conducted multiplex immunofluorescence analysis of pre‐treatment tissue samples. iCAF tumor infiltration (CFD, SLIT2, FAP, and COL14A1 as markers) was enhanced in non‐responders compared with responders (Figure 6a,b). CFD, SLIT2, FAP, and COL14A1 expression in tumor tissues significantly differed among patients with varying levels of TRGs (Figure 6c–f). Kaplan‐Meier analysis also showed that the positive cell percentages (PCP) of COL14A1, FAP, SLIT2, and CFD were significantly associated with worse prognosis in patients with LAGC undergoing immunochemotherapy (Figure 6g.h).

Meanwhile, eCAF tumor infiltration (POSTN and CXCL14 as markers) was enhanced in non‐responders compared with responders (Figure Sa,b). POSTN and CXCL14 expression in tumor tissues significantly differed among patients with varying levels of TRGs (Figure S7c, Supporting Information). Further, POSTN and CXCL14 have a strong co‐localization relationship, which might suggest that these two factors are associated with eCAF cells. Kaplan‐Meier analysis also showed that the PCP of POSTN and CXCL14 were significantly associated with better prognosis in patients with LAGC undergoing immunochemotherapy (Figure S7f,g, Supporting Information).

### 2.8 iCAF and eCAF Mediated a Differential Modulation of T Cell Function within Gastric Cancer Tumors

**Figure 7:**
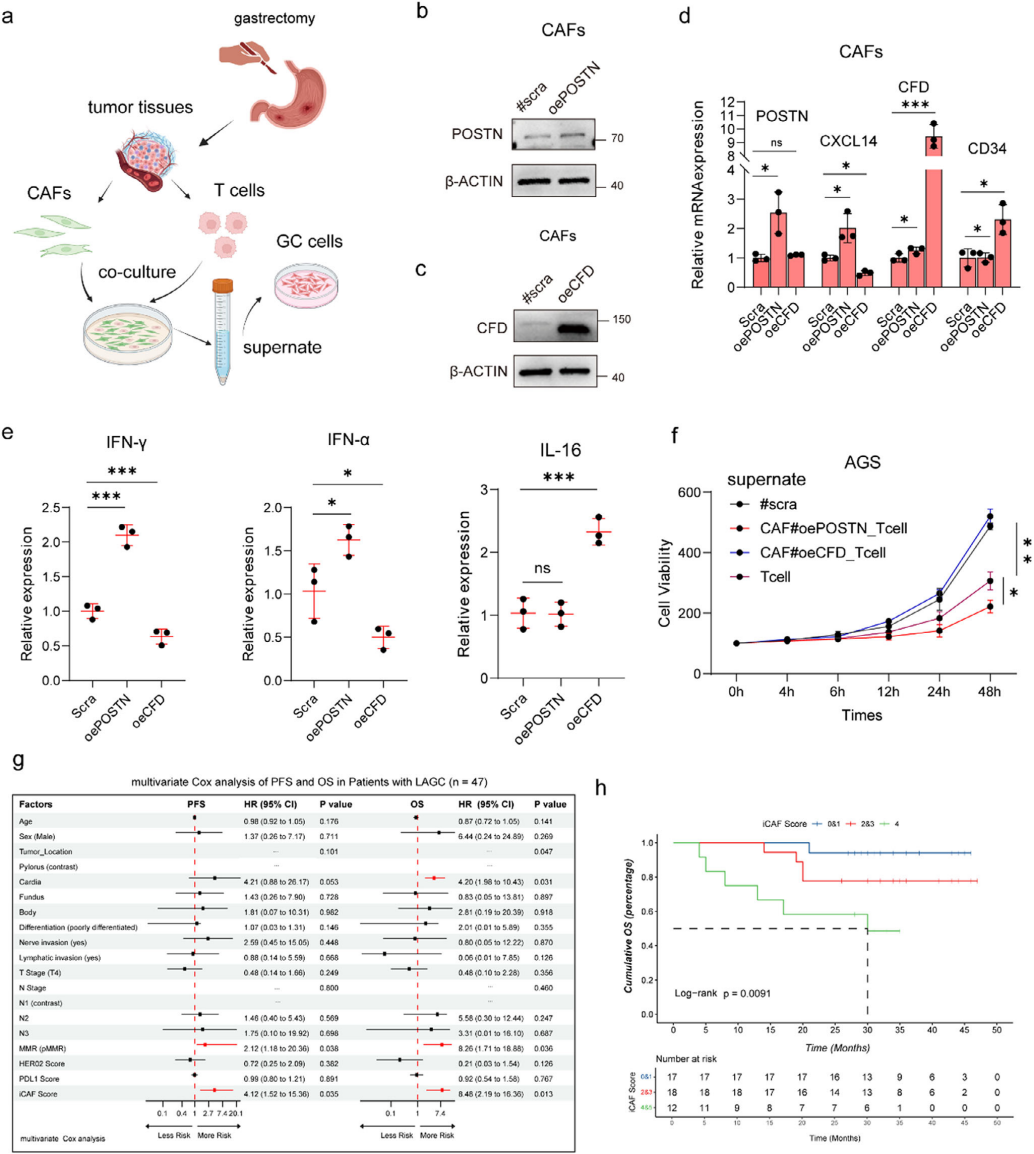
CAFs can activate or inhibit the functions of T cells. a) Schematic diagram showing the co‐culture system of CAFs, T cells, and GC cells. b,c) Western blot validation of POSTN and CFD expression in CAFs after gene overexpression. d) Relative mRNA expression of POSTN, CXCL14, CD34, and CFD in CAFs following POSTN or CFD overexpression. e) Quantification of cytokine expression (IFN‐γ, IFN‐α, and IL‐16) in T‐cell supernatants after co‐culture with CAFs. f) Cell viability assay of AGS GC cells treated with supernatants from different T‐cell co‐cultures (CAF#oePOSTN, CAF#oeCFD, or control). g) Multivariate Cox regression analysis of PFS and OS in patients with LAGC receiving immunotherapy. h) Kaplan–Meier analysis of OS based on iCAF scores in patients with LAGC. PFS, progression‐free survival; OS, overall survival; CAF, cancer‐associated fibroblast; iCAF, inflammatory CAF; GC, gastric cancer. ^*^ *p* < 0.05, ^**^ *p* < 0.01, ^**^ *p* < 0.001.

To further validate the distinct interactions between iCAF/eCAF and immune cells, we established primary CAF cell lines overexpressing POSTN and CFD (**Figure** **7**a). Successful construction of the iCAF and eCAF models was confirmed by Western blot (WB) and qPCR (Figure 7b–d). We then performed co‐culture experiments of iCAF or eCAF with T cells and analyzed cytokine levels in the supernatant (Figure S8a, Supporting Information). The results showed significantly elevated levels of IFN‐γ and IFN‐α in the eCAF–T cell co‐culture system, whereas IL‐16 was increased in the iCAF–T cell co‐culture system (Figure 7e). To assess functional impact, the co‐culture supernatants were added to gastric cancer AGS cells, and proliferation was measured using CCK‐8 assays. The data indicated that iCAF suppressed the cytotoxic activity of T cells, whereas eCAF enhanced it (Figure 7f). Collectively, these in vitro findings demonstrate the dualistic role of CAFs in the immune microenvironment, which needs further exploration (Figure S8b, Supporting Information).

### 2.9 iCAF Infiltration Score Predicted PD‐1 Blockade Treatment Efficacy

To determine whether iCAF infiltration could predict the effect of immunotherapy, we defined the infiltration score of iCAFs as the PCP of COL14A1 (high: PCP > 20%, score = 1), FAP (high: PCP > 45%, score = 1), SLIT2 (high: PCP > 60%, score = 1) and CFD (high: PCP >30%, score = 1) regarding averages as cut‐off values, and calculated the area under the receiver operating characteristic curve (AUC). The AUC of the iCAF score was 0.914, higher than that of the PD‐L1 score (AUC = 0.630) (Figure S8d, Supporting Information). We conducted a multivariate Cox analysis of PFS and OS in patients with LAGC who received immunochemotherapy. Multivariate analysis revealed an iCAF score hazard ratio of 4.12 for PFS (95% confidence interval: 1.52–15.36, P = 0.035) and 8.48 for OS (95% confidence interval: 2.19–16.36, P = 0.013) (Figure 7g). Kaplan–Meier analysis indicated that patients with LAGC with higher iCAF scores had worse PFS (log‐rank P = 0.017) and OS (log‐rank P = 0.009) (Figure 7h; Figure S7c, Supporting Information). The 3‐year OS calibration curves showed a satisfactory degree of fit for predicting the prognosis of patients with LAGC treated with immunochemotherapy (Figure S8e,f, Supporting Information).

## 3 Discussion

Neoadjuvant immunotherapy has achieved considerable success in treating solid tumors, such as lung, colorectal, and renal cancers.^[^ ^29^, ^30^, ^31^ ^]^ However, patients with gastric cancer exhibit a lower therapeutic response to immunotherapy. Our previous work highlighted the weak predictive performance of traditional markers for LAGC prognosis with immunotherapy.^[^ ^32^ ^]^ This novel 3‐year follow‐up data revealed a median follow‐up period of 32 months (quartiles: 28–43 months), with median PFS and OS not reached. The 3‐year PFS and OS rates were 64.1% and 73.2%, respectively. Analysis of the scRNA‐seq dataset combined with the 3‐year follow‐up results showed that CAFs play an important role in immunotherapy. Three distinct CAF types were identified in LAGC, with iCAFs suppressing T‐cell immune cytotoxicity and fostering an immunosuppressive microenvironment. iCAF infiltration was associated with a worse prognosis in patients with LAGC. A predictive score based on iCAF infiltration scores was developed for individualized survival predictions in immunotherapy.

Substantial developments in tumor immunotherapy have occurred in the last decade.^[^ ^14^ ^]^ Studies such as KEYNOTE‐059^[^ ^9^ ^]^ KEYNOTE‐062,^[^ ^33^ ^]^ and ATTRACTION‐04^[^ ^34^ ^]^ demonstrated that chemotherapy plus nivolumab significantly extended disease‐free survival and increased objective remission rates of advanced metastatic gastric cancer compared to chemotherapy alone. More clinical studies have focused on neoadjuvant immunotherapy for G/GEJ cancers. An interim analysis of this study revealed that neoadjuvant and adjuvant pembrolizumab significantly improved pCR compared to placebo. Li et al. reported a neoadjuvant immunochemotherapy phase II study (NCT04341857) with high pCR (17.2%) and MPR (55.2%), and an R0 resection rate of 93.1%.^[^ ^35^ ^]^ The DRAGON IV^[^ ^36^ ^]^ trial showed a significantly higher pCR rate in the camrelizumab plus SOX group (18.3%) than with SOX alone (5.0%). Another phase II study (NCT03448835) reported that patients with G/GEJ tumors (n = 21) received neoadjuvant treatment with atezolizumab, followed by atezolizumab plus docetaxel, oxaliplatin, and capecitabine. The results showed high rates of pCR (45%) and MPR (70%), with 13/14 responders alive and disease‐free, and 5/6 dead owing to recurrence.^[^ ^7^ ^]^

Similarly, historical studies of neoadjuvant SOX chemotherapy alone have reported pCR rates of ≈5–16%, whereas the tislelizumab plus SOX regimen in our study achieved a pCR rate of 26.5%. This notable improvement highlights the incremental clinical benefit conferred by the addition of PD‐1 blockade to standard chemotherapy in locally advanced gastric cancer. Besides, we reported an MPR rate of 49.0%, with 19/24 responders alive and disease‐free, and 8/25 with disease recurrence. Although the above results indicate that neoadjuvant immunotherapy is clinically feasible in patients with LAGC, the follow‐up data of the KEYNOTE‐585^[^ ^13^ ^]^ study indicated that neoadjuvant and adjuvant pembrolizumab plus chemotherapy failed, revealing heterogeneity in immunotherapy outcomes. These results highlight the need for identifying novel biomarkers for patients with LAGC undergoing immunotherapy.

Studies have explored the intrinsic resistance mechanisms of CAFs in cancer.^[^ ^20^, ^25^, ^37^ ^]^ Previous studies have investigated similarities in CAF heterogeneity across cancer cells. Different CAF subtypes play different roles in GC tissues, such as ECM remodeling,^[^ ^38^ ^]^ regulating ferroptosis,^[^ ^39^ ^]^ progression,^[^ ^40^, ^41^ ^]^ and metastasis.^[^ ^42^ ^]^ An increasing number of studies focus on the association between CAFs and immune cells during immunotherapy.^[^ ^17^, ^43^ ^]^ Additionally, CAF heterogeneity plays an important role in the tumor immune microenvironment. Previous studies have reported that some CAF subtypes, such as myCAFs, iCAFs, and antigen‐presenting CAFs (apCAFs) have immunoregulatory capabilities.^[^ ^44^, ^45^ ^]^ apCAFs reflect the functional attributes of APCs, originating from mesenchymal cells; they are characterized by the expression of major MHC II molecules^[^ ^46^ ^]^ and can induce the expansion of regulatory T‐cells.^[^ ^47^, ^48^ ^]^ eCAFs exhibit high expression of POSTN. A previous study reported the definition of eCAFs, demonstrating that they promote cancer cell invasion in patients with GC.^[^ ^20^ ^]^ Our study unexpectedly found that eCAFs exhibited high expression of MHC I molecules rather than MHC II, differing from apCAFs. Additionally, eCAF promotion of CD8+ T‐cell infiltration was distinctive and different from the function of antigen presentation to CD4+ T‐cells by apCAFs. We believe these findings are interesting and worthy of exploration in future studies. iCAFs and myCAFs were more enriched in the tumors of responders than in those of non‐responders and contributed to evading PD‐1 blockade. We identified IL16 gene upregulation and demonstrated the expression of inflammatory factors, chemokines, and ECM components. Infiltration of iCAFs in non‐responders was confirmed using markers such as FAP, SLIT2, COL14A2, and CFD. Spatial transcriptomics further substantiated the immunosuppressive effect of iCAF in GC. Finally, we calculated the PCP of iCAF markers (FAP, SLIT2, COL14A2, and CFD) and determined their association with iCAF scores and survival of patients with LAGC. A score based on iCAF markers was developed to predict immunotherapy effects.

The perioperative tislelizumab plus SOX regimen demonstrated an acceptable and manageable safety profile. Most TRAEs were mild to moderate (Grade 1–2) and were effectively controlled with standard supportive measures, aligning with the safety data reported for other PD‐1 inhibitor‐based neoadjuvant regimens in gastric cancer. Hematologic toxicities, including myelosuppression, white blood cell decreased, and anemia, were the most common events and were consistent with the expected effects of oxaliplatin‐ and S‐1‐based chemotherapy. irAEs were infrequent and predominantly low‐grade, with rash being the most typical manifestation. A single case of HLH was observed, representing a rare but potentially fatal immune‐mediated complication associated with checkpoint blockade. This finding underscores the necessity for heightened clinical vigilance and prompt multidisciplinary management when immune‐related toxicity is suspected. Taken together, these findings suggest that the integration of tislelizumab into the perioperative setting is feasible and does not substantially exacerbate treatment‐related toxicity. The safety profile observed in this study supports further exploration of PD‐1‐based combination strategies to optimize the therapeutic window between efficacy and tolerability in locally advanced G/GEJ adenocarcinomas.

The spatial distribution of CAF subsets in GC was validated using a multi‐staining registration analysis. This confirmed the chemotactic ability of eCAFs to attract T‐cells. These findings were further confirmed via follow‐up of patients with GC in clinical trials. This study has several limitations. First, the sample size was relatively small, and further validation in larger, multicenter cohorts is required to confirm the robustness of our findings. Second, the tissue digestion process might have affected the integrity of the cellular transcriptome, which could influence single‐cell analysis results. Third, although the spatial distribution of CAF subsets in gastric cancer was validated using multi‐staining registration analysis, isolating and enriching other CAF subsets remained challenging due to limited sample availability, potentially introducing selection bias. Finally, the predictive score based on the iCAF score was developed using data from this trial, and its prognostic performance has not yet been confirmed in an independent external validation cohort. Future studies with larger and more diverse populations are warranted to externally validate and refine this model for broader clinical application.

## 4 Conclusion

Our study confirmed that adding tislelizumab to neoadjuvant SOX therapy demonstrates promising efficacy and safety in patients with locally advanced G/GEJ adenocarcinomas. Approximately 37000 cells were profiled from the tumor tissues of eight patients with GC using scRNAseq. These results indicate that CAFs play an important role in GC immunotherapy, and inhibiting iCAF activation may be a potential therapeutic target for patients with GC undergoing immunotherapy.

## 5 Experimental Section

### Study Design and Participants

This was a prospective single‐arm phase II trial (ClinicalTrials.gov: NCT04890392), approved by the Institutional Review Board of Union Hospital, Tongji Medical College, Huazhong University of Science and Technology (No. 2020‐0447), in accordance with the Declaration of Helsinki and Good Clinical Practice. All patients provided written informed consent. Patients received the study treatment free of charge without compensation.

This study was conducted at Union Hospital, Tongji Medical College, Huazhong University of Science and Technology in Wuhan, China between September 15th, 2020, and July 15th, 2022. Eligible patients were between 18 and 75 years old with histologically confirmed gastric and/or gastro‐oesophageal junction (G/GEJ) adenocarcinoma; a cT3/4aN+M0 tumor stage confirmed using contrast‐enhanced computed tomography (CT)/magnetic resonance imaging (MRI) of the chest, abdomen, and pelvis, and ultrasound gastroscopy, according to the 8th edition of the American Joint Committee on Cancer (AJCC) staging system; and an Eastern Cooperative Oncology Group performance status of 0–1. Patients with active or suspected autoimmune disease or distant metastases were excluded. The study protocol is provided in Supplementary Note 1. A total of 49 patients were enrolled. Figure 1a,b shows the patient selection flowchart.

### Treatment Procedures

Three cycles of neoadjuvant tislelizumab plus SOX were administered preoperatively. Eligible patients received 200 mg intravenous tislelizumab on the first day of every 21‐day cycle. SOX chemotherapy consisted of oral S‐1 (40 mg twice a day) based on body surface area (<1.25 m^2^, 80 mg d^−1^; ≥1.25 to <1.5 m^2^, 100 mg d^−1^; ≥1.5 m^2^, 120 mg d^−1^) from days 1 to 14. Oxaliplatin was administered intravenously (130 mg m^−2^) on day 1 of each 21‐day cycle. Dose adjustments were not performed for tislelizumab. Dose decreases for S‐1 and oxaliplatin were authorized depending on adverse events (AEs) during neoadjuvant/adjuvant treatment. Radical gastrectomy with D2 lymph node dissection was performed within 4–6 weeks of neoadjuvant therapy completion in patients without tumor progression via preoperative imaging assessment. Adjuvant chemotherapy with SOX at the same dosages was recommended for five cycles, starting 4–8 weeks after surgery. If disease progression, distant metastasis, or unacceptable toxicity occurred, the treatment regimen was discontinued, and the most appropriate alternative regimen was determined based on the patient's clinical status.

### Radiological and Pathological Assessments

Radiological assessment was performed using the Response Evaluation Criteria in Solid Tumors version 1.1 with MRI or CT at baseline, after two cycles, before surgery, every 6 months after surgery for 3 years, and annually thereafter. Tumor staging after surgery, followed by neoadjuvant therapy (post‐neoadjuvant pathologic TNM Classification of Malignant Tumors), was performed according to the AJCC Staging System, 8th ed.

Pathological responses were evaluated by an independent pathologist. Surgical specimens were stained with hematoxylin and eosin and analyzed to determine the percentage of residual viable tumor cells. A complete pathological response (CPR) was defined as the absence of viable tumor cells. A major pathological response (MPR) was defined as the presence of ≤10% viable tumor cells. The tumor regression grade (TRG) (a modified Ryan scheme) was classified as follows: TRG 0 (CPR in the primary tumor and lymph nodes, no residual tumor cells), TRG 1 (near‐complete response, ≤10% residual tumor cells), TRG 2 (partial response, 10–50% residual tumor cells), and TRG 3 (poor or no response, ≥50% residual tumor cells).

### Treatment‐Related Adverse Events Assessment

Treatment‐related adverse events(TRAEs) were assessed and graded according to the Common Terminology Criteria for Adverse Events (CTCAE, version 5.0) from the initiation of neoadjuvant or adjuvant therapy until 28 days after the last dose, or until resolution if ongoing. Perioperative morbidity and mortality were prospectively recorded. Postoperative surgical complications were evaluated according to the Clavien–Dindo classification system. Immune‐related adverse events (irAEs) were identified based on clinical presentation and laboratory findings, and graded using CTCAE v5.0.

### Single‐Cell RNA‐Seq Data Processing

In‐house FASTQ files generated from 10× Genomics were aligned and quantified against the GRCh38 human reference genome using Cell Ranger (version 6.1.2) with default settings. The output of Cell Ranger and the count matrix were read using *Read10×* from the *Seurat* package (version 4.0.4) and *read.table*, respectively. The latter was further converted to dgCMatrix format. Potential doublets predicted using Scrublet were removed.^[^ ^49^ ^]^ *The merge* function was used to aggregate individual objects, and *RenameCells* was used to ensure that all cell labels were unique. In total, 28 378 cells from GC tissues of different patients with immunotherapy were pooled. Quality control was applied based on several criteria. Briefly, cells with <200 detected genes and >20% mitochondrial content were excluded. Cells with>6000 detected genes were eliminated to exclude possible doublets. After filtering, 22248 high‐quality cells were preserved for subsequent analyses in patients with immunotherapy.

### Single‐Cell RNA‐Seq Data Merge and Normalization

Global‐scaling normalization (“LogNormalize”) was employed to ensure that the total gene expression in each cell was equal (scale factor = 10000). The top 2000 variably expressed genes were returned for downstream analysis using *FindVariableFeatures*. *ScaleData* with the “vars.to.regress” option for UMI and percent mitochondrial content were used to regress out unwanted sources of variation. Principal component analysis incorporating highly variable features reduced dimensionality, and the first 30 principal components were identified for analysis. *RunFastMNN* in the *SeuratWrappers* package (version 0.3.0) was used for batch correction. Clustering analysis was performed based on edge weights between any two cells, and a shared nearest‐neighbor graph was produced using the Louvain algorithm implemented in *FindNeighbors* and *FindClusters*. Identified clusters were visualized using the UMAP method. A similar procedure was applied for CAF subclustering. To annotate the cell clusters, differentially expressed markers were identified using *FindAllMarkers* with the default nonparametric Wilcoxon rank‐sum test and Bonferroni correction.

### Cell‐Cell Interaction Analysis

CellphonedDB^[^ ^50^ ^]^ was used to analyze cell‐cell interactions among TME components. Input files consisted of a raw count matrix extracted from the Seurat object and an annotation file of cell types. The heatmap_plot function from CellphoneDB and the *Circlize* package (version 0.4.14) were used to display interaction frequency between cell subsets. Visualization of the potential interaction strength between the ligand and receptor, predicted based on average expression, was performed using *dot_plot* and *pheatmap* package (version 1.0.12). Significant ligand–receptor pairs (P<0.01) were extracted for illustration.

### Enrichment Analysis

Pseudotime‐dependent genes were subjected to Gene Ontology (GO) and Kyoto Encyclopedia of Genes and Genomes (KEGG) enrichment analyses using the *clusterProfiler* package (version 3.0.4) with default settings. A fifty hallmark gene set in the MSigDB database (https://www.gsea‐msigdb.org/gsea /msigdb) was used for gene set enrichment analysis (GSEA) of iCAF and eCAF clusters using the *eSCAPe* package (version 1.4.0).

### Spatial Transcriptomics

Raw base‐call files were converted to FASTQ reads using bcl2fastq. A total of 29808 locations in nine primary GC samples were analyzed, and the spatial transcriptomics data yielded 1882–4274 spots per histological section in each of the nine cases (median: 3491 spots) in GSE251950. Read10X_h5 and *CreateSeuratObject* from the *Seurat* package were used to create an object using the Space Ranger outfit. *Read10X_Image* was used to load hematoxylin and eosin image data. The dataset was normalized using *logNormalize*. *SpatialFeaturePlot* showed the expression level of a single gene at a spatial location. Spots in which the expression of genes in immune pathways and CAF were simultaneously non‐zero were recolored, with the intensity reflecting the average expression level. The *AUCell* package was used to calculate scores of immune pathways and CAF infiltration.

### Multiplexed Immunofluorescence Analysis

All surgical tissue samples were processed into formalin‐fixed paraffin blocks and cut into 5‐micrometer‐thick sections. Multiplex immunohistochemical (IHC) staining was performed using the Opal 5‐color kit (Akoya Bioscience, NEL801001KT) to evaluate CFD (GB12045, Servicebio, 1:5000, Opal 440), COL14A1 (AF0573, Affinity Biosciences, 1:2000, Opal 488), FAP (11779‐1‐AP, 1:2000, Opal 555), SLIT2 (20217‐1‐AP, ProteinTech, 1:2000, Opal 647), CXCL14 (10468‐1‐AP, ProteinTech, 1:1000, Opal 647) and POSTN (66491‐1‐Ig, 1:1000, Opal 488). Briefly, sections were dewaxed with xylene for 20 min and rehydrated with ethanol. Microwave treatment was performed for antigen retrieval using a buffer (pH 9.0). All sections were cooled to room temperature for 30 min. Endogenous peroxidase activity was blocked using an antibody diluent/block (72 424 205; Akoya Bioscience) at room temperature for 10 min. Slides were incubated with a primary antibody at room temperature for 1 h, followed by secondary reagents at 37 °C for 20 min, and tyramide signal amplification reagents at room temperature for 10 min (Opal 480, Opal 520, Opal 620, and Opal 690; Akoya Bioscience, 1:100). MWT antigen retrieval was performed until all markers were stained. Nuclear staining was performed using DAPI (Akoya Bioscience, 1:5) at room temperature for 5 min. Slides were mounted using anti‐fade fluorescence mounting medium (ab104135; Abcam) and stored at 4 °C until image acquisition. Slides were scanned using a PerkinElmer Vectra Polaris (PerkinElmer) and a confocal microscope (TCS SP8, Leica). The percentage of stained cells among all nucleated cells was determined. Multispectral image unmixing was performed using QuPath (version 3.0) and ImageJ (version 1.53i). Briefly, DAPI‐positive cells were identified using the “cell detection” command, and each channel threshold was selected. All detected cells were divided into different subgroups for further analysis, and defective samples or areas with staining artifacts were reanalyzed or excluded.

### Isolation and Culture of Cancer‐Associated Fibroblasts (CAFs)

Primary CAFs were isolated from freshly resected gastric tumor tissues obtained from patients undergoing surgery at Union Hospital, Tongji Medical College, Huazhong University of Science and Technology, with written informed consent and approval from the institutional ethics committee (Approval No. 2020‐0447). Tumor specimens were rinsed with phosphate‐buffered saline (PBS) and minced into 1–2 mm^3^ fragments. The tissue was digested with 0.1% collagenase type I (Sigma‐Aldrich) and 0.05% DNase I at 37 °C for 1 h with gentle agitation to ensure optimal enzymatic digestion. The cell suspension was filtered through a 70 µm cell strainer and centrifuged at 300 × g for 5 min. The pellet was resuspended in Dulbecco's modified Eagle's medium (DMEM; Gibco) supplemented with 10% fetal bovine serum (FBS) and 1% penicillin–streptomycin, and plated in T25 flasks. After 24–48 h, non‐adherent cells were removed by PBS washing. CAFs were maintained at 37 °C in a humidified incubator with 5% CO_2_, and cells between passages 3–8 were used for all experiments. The fibroblast phenotype was confirmed by immunofluorescence staining for α‐smooth muscle actin (α‐SMA) and vimentin and by the absence of cytokeratin expression.

### Isolation and Culture of Primary T Cells

Primary CAFs were isolated from freshly resected gastric tumor tissues obtained from patients undergoing surgery at Union Hospital, Tongji Medical College, Huazhong University of Science and Technology, with written informed consent and approval from the institutional ethics committee (Approval No. 2020‐0447). Peripheral blood mononuclear cells (PBMCs) were isolated by density gradient centrifugation using Ficoll‐Paque PLUS (GE Healthcare). CD3⁺ T cells were purified from PBMCs by magnetic‐activated cell sorting (MACS) using a Pan T Cell Isolation Kit (Miltenyi Biotec) according to the manufacturer's instructions. The purity of isolated T cells (>95%) was confirmed by flow cytometry.

Purified T cells were cultured in RPMI‐1640 medium supplemented with 10% FBS (Gibco), 1% penicillin–streptomycin, and 100 U/mL recombinant human IL‐2 (PeproTech). IL‐2 was replenished every 48 h to maintain T‐cell viability. For activation, T cells were stimulated with anti‐CD3/CD28 Dynabeads (Thermo Fisher Scientific) at a 1:1 bead‐to‐cell ratio and incubated at 37 °C in 5% CO_2_. Activated T cells from the same donor were used across experimental groups to minimize donor variability.

### Generation of POSTN‐ and CFD‐Overexpressing CAFs

CAFs were transduced with lentiviral vectors encoding human POSTN or CFD (GeneChem, Shanghai, China) or with an empty control vector. Transduction was performed at a multiplicity of infection (MOI) of 10 in the presence of 8 µg mL^−1^ polybrene. After 24 h, the medium was replaced, and cells were selected with puromycin (2 µg mL^−1^) for 72 h to establish stable overexpression lines. Transduction efficiency was verified by qPCR and western blotting prior to use in co‐culture experiments.

### Co‐Culture of CAFs and T Cells

To assess the effects of CAF‐derived POSTN and CFD on T‐cell function, three experimental groups were established: POSTN‐overexpressing CAFs (CAFs‐POSTN): CAFs transfected to overexpress POSTN were directly co‐cultured with activated T cells. CFD‐overexpressing CAFs (CAFs‐CFD): CAFs overexpressing CFD were directly co‐cultured with activated T cells. Control group (CAFs‐Ctrl): untransfected CAFs were co‐cultured with activated T cells. CAFs were seeded in 6‐well plates (2 × 10⁵ cells/well) and allowed to adhere overnight. Activated T cells were then added at a 1:1 ratio (T cells:CAFs) for direct contact co‐culture. The co‐cultures were maintained in RPMI‐1640 medium containing 10% FBS for 48–72 h. After co‐culture, the culture supernatants were collected for cytokine analysis.

### IFN‐γ and IFN‐α, IL‐16 Enzyme‐Linked Immunosorbent Assay (ELISA)

The concentration of IFN‐γ and IFN‐α, IL‐16 in culture supernatants was determined using a Human ELISA Kit (R&D Systems) according to the manufacturer's protocol. Briefly, 100 µL of standards or diluted samples were added to antibody‐coated 96‐well plates and incubated for 2 h at room temperature. After washing, wells were incubated sequentially with a biotinylated detection antibody (1 h) and streptavidin–HRP (30 min). The TMB substrate solution was then added, and the reaction was stopped with 2 N H_2_SO_4_. Absorbance was measured at 450 nm with reference to 570 nm using a microplate reader (BioTek). Concentrations were calculated from a standard curve generated with recombinant proteins.

### Survival Analysis

The Kaplan–Meier analysis inferred CAF infiltration scores from available mIHC data in this immunotherapy trial with detailed follow‐up information. The algorithm was run with a web portal auto‐generated signature matrix and 1000 permutations. *Survival* (version 2.42–3) and *Survminer* (version 0.4.9) packages were used for analysis and visualization.

### Endpoints

The primary endpoint was the MPR rate. Secondary endpoints included the pCR rate, disease control rate (DCR), PFS, OS, and safety. DCR was defined as the percentage of patients whose intervention led to a pCR, partial response, or stable disease. PFS was the period from enrolment to the first occurrence of disease progression, recurrence, or all‐cause death, whichever occurred first. OS was the period from enrolment to all‐cause death.

### Statistical Analyses

Continuous data are presented as medians with interquartile ranges or ranges. Categorical data are summarized as frequencies (percentages) and compared using the chi‐square test. PFS and OS were estimated using the Kaplan–Meier method. The log‐rank test determined survival differences across subgroups. The median follow‐up period was calculated using the reverse Kaplan–Meier method. Efficacy and safety were evaluated. Continuous variables were transformed into categorical variables using optimal cutoff values. Univariate and multivariate analyses were performed using Cox regression analysis. Variables with *p* < 0.05 in univariate analysis were included in the multivariable analysis. Variables with *p* < 0.05 in the multivariate Cox analysis of OS were selected as parameters in the score constructed based on the results of the multivariable Cox regression analysis. Hosmer–Lemeshow goodness‐of‐fit tests evaluated discrimination and calibration. All statistical tests were performed using R (version 4.0.1; Auckland, New Zealand) and Prism 8 (version 8.02; Charlotte, NC, USA). A two‐sided P<0.05 was considered statistically significant.

### Ethics Approval and Consent to Participate

This study (No. 2020‐0447) was approved by the Ethics Committee of Union Hospital, Tongji Medical College, Huazhong University of Science and Technology. Participants gave informed consent to participate in the study before taking part.

## Conflict of Interest

The authors declare no conflict of interest.

## Author Contributions

Y.L., X.S., and C.L. are co‐first authors and contributed equally to this study. Y.P.Y. is the guarantor of our research. K.X.T., Y.P.Y., and J.S.F. conceived and designed the study. X.S. and K.W. were responsible for patient recruitment. G.B.W., Z.W., and K.L.C. were responsible for patient treatment and patient care. A.S.L. and K.L. were responsible for the surgery. M.Y. and X.M.S. helped with pathological evaluation. M.Y. helped with image evaluation. P.Z. was responsible for quality control of treatment. Y.L. collected the data. Y.L., X.S., and C.G.L. analyzed and interpreted clinical data. Y.L. and X.S. were responsible for biomarker analysis. Y.L. wrote the first draft of the manuscript.

## Supporting information



Supporting Information

Supporting Information

## Data Availability

The data that support the findings of this study are available from the corresponding author upon reasonable request.
