# Supplementary material for: Three‐Year Follow‐Up of Neoadjuvant Tislelizumab with Chemotherapy in Locally Advanced Gastric and Gastroesophageal Junction Adenocarcinoma: Revealing Cancer‐Associated Fibroblast Heterogeneity Corresponding to PD‐1 Blockade Efficacy
Source: Adv Sci (Weinh). 2025 Dec 1;13(9):e08433. doi: 10.1002/advs.202508433 (PMC12903965; doi:10.1002/advs.202508433)
Supplement: Supplementary file 1 — Supporting Information [file ADVS-13-e08433-s002.docx]

**Supplement Figures**

**Figure S1** **(a)** Dot plot showing the expression of representative marker genes in different cell types from patients receiving immunotherapy. **(b)** Dot plot showing the expression of representative marker genes in different cell types from patients without immunotherapy. **(c)** Proportions of fibroblasts in tumor and adjacent tissues, and between responders and non-responders. **(d)** UMAP visualization of single-cell clusters in non-responders and responders. **(e)** CopyKAT analysis showing diploid and aneuploid cell distributions in non-responder and responder patients. ns, not significant; *p < 0.05; **p < 0.01.

**Figure S2 (a)** UMAP depicting clusters of CAFs in LAGC tissues. **(b)** Heatmap depicting marker genes associated with CAF subtypes. **(c)** Dot plot showing the average expression and percentage of cells expressing representative CAF markers. **(d)** UMAP feature plots showing the expression of key marker genes (MYH9, COL1A1, MYO16, CCL19, CD34, CFD, POSTN, and CXCL14) across CAF subtypes. **(e-g)** GSEA of eCAFs, iCAFs, and myCAFs. CAF, cancer-associated fibroblast; iCAF, inflammatory CAF; eCAF, extracellular matrix-related CAF; myCAFs, myofibroblastic CAF; LAGC, locally advanced gastric cancer; GSEA, gene set enrichment analysis.

**Figure S3 (a)** Violin plots showing the expression levels of C3, C7, CFH, CFD, and CD34 across CAF subtypes. **(b)** Violin plots showing the expression levels of COL14A1, FAP, SLIT2, and ACTA2 across CAF subtypes. **(c)** Violin plots showing the expression levels of MUC1, MUC5AC, COL4A5, and COL6A2 across CAF subtypes. **(d)** Violin plots showing the expression levels of ADAM28, POSTN, and CXCL14 across CAF subtypes. CAF, cancer-associated fibroblast.

**Figure S4 (a)** Dot plots showing the expression levels of MHC I (HLA-A, HLA-B, HLA-C) and MHC II (CD74, HLA-DRB1, HLA-DRA, HLA-DQA1, HLA-DPA1) genes across CAF subtypes. **(b)** Comprehensive analysis of ligand–receptor interactions between different CAF subtypes and other cell types in responders and non-responders. **(c)** Heatmaps illustrating the ligand–receptor interactions involved in the top four signaling pathways among CAF subtypes and other cell types. CAF, cancer-associated fibroblast; MHC, major histocompatibility complex.

**Figure S5 (a)** UMAP visualization of spatial transcriptomic spots from samples in the GSE251950 dataset. **(b)** Dot plots showing the expression of representative marker genes for major cell types across all samples. **(c)** Spatial maps showing the distribution of different cell type niches in STAD#1, STAD#3, STAD#4, STAD#6, STAD#7, STAD#8, and STAD#9. CAF, cancer-associated fibroblast; STAD, stomach adenocarcinoma.

**Figure S6 (a)** Bar plots showing the top 30 marker genes of iCAFs and eCAFs ranked by log fold change and statistical significance. **(b)** Comparison of AUcell Z-scores for iCAFs, eCAFs, MHC I pathway, CD8⁺ T cells, T-cell co-stimulation, and inflammation-promoting signatures between immunogenic and non-immunogenic GC samples. **(c)** Violin plots showing the AUcell scores of the MHC I pathway in STAD#2 and STAD#5. **(d)** Spatial maps displaying the distribution of the MHC I pathway in STAD#2 and STAD#5. **(e)** Violin plots showing the AUcell scores of eCAFs in STAD#2 and STAD#5. **(f)** Spatial maps displaying the spatial distribution of eCAFs in STAD#2 and STAD#5. *p < 0.05, **p < 0.01, **p < 0.001. CAF, cancer-associated fibroblast; iCAF, inflammatory CAF; eCAF, extracellular matrix-related CAF; GC, gastric cancer; AUcell, Area Under the Curve cell scoring.

**Figure S7 (a, b)** Multiplex immunofluorescence staining of POSTN and CXCL14 in LAGC tissues from a responder and a non-responder. Tumor and adjacent regions are indicated by dashed lines. **(c)** Quantification of POSTN⁺ and CXCL14⁺ cells in patients with different TRGs. **(d, e)** Representative images and co-localization analysis of POSTN and CXCL14 expression in LAGC tissues. The right panel shows fluorescence intensity profiles and correlation (R² = 0.354, p < 0.001). **(f, g)** Kaplan–Meier analysis of OS and PFS according to POSTN/CXCL14 co-expression levels in patients with LAGC treated with immunotherapy. PCP, percentage of positive cells; OS, overall survival; PFS, progression-free survival; TRG, tumor regression grade; LAGC, locally advanced gastric cancer. *p < 0.05, **p < 0.01, **p < 0.001.

**Figure S8 (a)** Representative microscopic image of the co-culture of CAFs and T cells. **(b)** Schematic illustration showing how iCAFs and eCAFs modulate T-cell activity in the tumor microenvironment through IL-16 secretion, MHC I expression, and IFN-γ/IFN-α signaling. **(c)** Kaplan–Meier curve of PFS in LAGC patients stratified by iCAF score. **(d)** ROC curves comparing the predictive performance of iCAF score, HER2, and PD-L1 for MPR. **(e, f)** Calibration curves evaluating the accuracy of the iCAF-based model in predicting 3-year PFS and OS. CAF, cancer-associated fibroblast; iCAF, inflammatory CAF; eCAF, extracellular matrix-related CAF; MPR, major pathologic response; PFS, progression-free survival; OS, overall survival; ROC, receiver operating characteristic. *p < 0.05; **p < 0.01; **p < 0.001.
